# Critical evaluation of the benefits and limitations of foam posturography in vestibular disorders: a narrative review

**DOI:** 10.3389/fneur.2026.1771719

**Published:** 2026-02-25

**Authors:** Mitesh Patel, Carl Walter, Per-Anders Fransson

**Affiliations:** 1 North Wales Medical School, Bangor University, Bangor, United Kingdom; 2 Department of Otorhinolaryngology, Head and Neck Surgery, Skåne University Hospital, Lund, Sweden; 3 Department of Clinical Sciences Lund, Lund University, Lund, Sweden

**Keywords:** applications, clinical utility, diagnostic sensitivity, foam, methodological variability, performance, posturography

## Abstract

This review synthesises current evidence on the clinical utility of foam-based posturography in the assessment of vestibular pathologies. It critically evaluates: (1) the physiological mechanisms underlying foam posturography and its capacity to determine vestibular contributions; (2) methodological variability across testing protocols, including differences in foam properties, stance conditions and measurement parameters; (3) the diagnostic sensitivity, specificity and reliability of foam posturography in various vestibular disorders; (4) its performance relative to other balance and vestibular assessments to determine reciprocity; and (5) its applications in rehabilitation. The review also highlights current gaps and challenges, proposing future directions aimed at standardising foam posturography protocols and strengthening their integration into routine clinical practice as part of comprehensive vestibular assessment. Foam posturography is a robust, reliable, and cost-effective method that alters the reliability and quality of somatosensory input to evaluate the role of vestibular function. It provides objective measures to support the diagnosis of vestibulopathies and distinguish between pathologies and monitor the results of rehabilitation. Limitations include non-standardised methodology and availability of normative data. However, its affordability and portability make it a practical and valuable adjunct in both clinical and research settings.

## Introduction

Postural control is a complex and dynamic process that relies on the integration of multisensory inputs from the vestibular, visual and somatosensory systems to maintain an upright position (1). The central nervous system (CNS) continuously processes and weights these sensory inputs to generate appropriate motor responses, allowing individuals to maintain stability during static stance, perturbed stance and dynamic movements (2–4). Disruption or impairment in any of these sensory modalities, and especially in the vestibular apparatus, can lead to significant deficits in balance, resulting in symptoms such as dizziness, vertigo, unsteadiness and an increased risk of falls (5–7). A clinical example of this is that patients with bilateral vestibular loss experience substantial postural instability when they close their eyes (8, 9).

### Sensory contributions to postural control

The vestibular system, comprising the semicircular canals and otolith organs, provides critical information about head position and motion relative to gravity (10). Visual input offers external spatial cues that help orient the body relative to objects in the environment, while somatosensory feedback from muscle spindles, joint receptors and cutaneous mechanoreceptors informs the CNS about body position and surface contact, texture and plantar-to-surface pressure (11). These sensory inputs are reweighted, wherein there is dynamic adjustment on the reliance of each modality according to environmental settings or sensory perturbations. For example, on unstable surfaces, such as standing on foam, visual and vestibular inputs gain prominence (12, 13), whereas somatosensory inputs are prioritised under stable surface conditions.

### Vestibular dysfunction and balance assessment

Vestibular dysfunction is a common cause of postural instability and falls, particularly in older adults and individuals with peripheral or central vestibulopathies (6, 14, 15). Accurate clinical assessment of vestibular function is crucial not only for diagnosis but also for guiding rehabilitation and monitoring recovery. Standard vestibular laboratory tests include caloric testing, Vestibular Evoked Myogenic Potentials (VEMPs) and video Head Impulse Testing (vHIT). While these tests, and others, quantify specific vestibular organ or reflex pathways, they provide limited information on the functional integration of sensory inputs for balance and may not fully capture the impact of vestibular deficits on postural control.

### Posturography and its role

Posturography encompasses a range of assessment techniques aimed at quantifying postural sway and stability under controlled sensory conditions. The Sensory Organization Test (SOT) (16), a component of computerised dynamic posturography (CDP), systematically manipulates visual and support surface conditions to evaluate the relative contributions and integration of sensory inputs during stance (17, 18). Despite its diagnostic precision, CDP is limited by high cost, bulky equipment and restricted accessibility. Foam posturography emerges as an accessible, cost-effective and clinically feasible alternative to CDP for assessing sensory contributions to balance, especially vestibular function. By having patients stand on a compliant foam surface, somatosensory feedback from the feet and ankles is distorted or attenuated, increasing reliance on vestibular and visual inputs. When combined with an eyes closed (EC) condition, this approach effectively increases the vestibular system’s role in maintaining postural stability. The foam Romberg test, a bedside adaptation, provides rapid qualitative insights by observing sway or falls during EC stance on a foam pad. However, modern protocols often incorporate quantitative measures, such as centre-of-pressure (CoP) metrics from force plates or stabilometers, to enhance objectivity and sensitivity.

The aim of this review is to critically analyse and synthesise current research on the clinical utility of foam-based posturography in the assessment of vestibular pathologies. The review specifically examines the physiological rationale that underpins foam posturography, evaluates methodological variability across different protocols and equipment and appraises its reliability in different vestibular disorders. In addition, foam posturography is considered in relation to other vestibular assessments to consider its role within a clinical evaluation. This review is timely given uncertainty on protocol standardisation, availability of normative data and the extent to which foam posturography can reliably distinguish vestibular disorder from healthy individuals. By consolidating current findings and identifying limitations and gaps, this review aims to inform clinicians and researchers about the use of foam posturography and considerations to support more consistent application across settings. This narrative review aims to guide future efforts in strengthening the validity of foam posturography and its integration into routine vestibular assessment.

### Scope and objectives of the review

This review synthesises current evidence on the clinical utility of foam-based posturography in vestibular pathologies. It critically examines: 1. The physiological basis underpinning foam posturography and its ability to reveal vestibular contributions; 2. Methodological variability in foam testing protocols, including foam material properties, stance conditions and measurement parameters; 3. Diagnostic sensitivity, specificity, and reliability of foam posturography in different vestibular disorders; 4. Comparisons with other tests to establish reciprocity; and 5. Rehabilitation applications. Finally, the review identifies existing gaps and challenges, proposing future directions aimed at standardising foam posturography protocols and enhancing their integration into routine clinical practice for comprehensive vestibular assessment.

## Clinical objectives of posturography

From a clinical perspective, posturography serves several objectives. It can be used to assess sensory reweighting, by quantifying how individuals modify postural control strategies when visual or somatosensory inputs are altered, thereby evaluating vestibular contributions to postural control. Posturography also provides an objective evaluation of functional performance, capturing subtle instabilities that may not be evident during routine examinations. In addition, posturographic measures are increasingly used to estimate fall risk, particularly in older adults and neurological or vestibular populations. Finally, posturography supports rehabilitation planning and monitoring, allowing customised therapy based on sensory dependence and objectively tracks changes in postural control over time.

Within the context of investigations, posturography is not deemed to be diagnostic. Other measures can ascertain the degree of vestibular function. Patients with dizziness or imbalance from suspected vestibular origins are generally examined by a neuro-otology specialist. A clinical history can lead a physician to suspected causes. However, vestibular syndromes can overlap. For example, about 14% of patients with suspected Meniere’s disease experience migraine (19, 20), which can make it difficult to identify the cause of vertigo or most problematic issue. The neuro-otology specialist will utilise various tests to decide whether the issue relates to the peripheral or central vestibular pathways which traditionally includes the vestibular caloric test and rotatory test of the semicircular canals. More recent developments have advocated the vestibular-evoked myogenic potential with or without the high-velocity video head-impulse test. However, as posturography measures how unsteady a person is in quiet standing or under different perturbing conditions, it provides a functional measure applicable to everyday activities and clinical outcomes with regard to fall risk and the outcomes of rehabilitation.

Vestibular pathologies are particularly complex, and patients may experience symptoms including dizziness, vertigo, imbalance, paraesthesia, hearing loss, photophobia, visual aura or visual dependency to name a few (21, 22). A postural imbalance may be deemed of lower value to other complaints by patients or clinicians initially, but postural control is linked to patient autonomy and well-being (23, 24). Future fall-risk can increase anxiety, and therefore, the long-term changes associated with postural instability are important to consider (25, 26). Postural adaptation has been studied as a technique that controllably perturbs balance of patients. This method establishes whether the patient is likely to be at substantial risk of future falls and contributes to identifying the most suitable mode of rehabilitation (3, 27–31). Although proprioceptive vibration can be employed to perturb balance, foam pads are cheaper and require less technical knowledge. A foam pad can be used to test adaptation between sensory conditions, such as between different visual perturbations. When asking a patient to stand on a foam pad, the reliability of somatosensory feedback is altered from the solid surface underneath the foam pad and the perturbation is imposed mechanically, as forces that maintain stability are dampened by the material (13, 32, 33).

Furthermore, the presentation of dizziness or imbalance may be acute, episodic or it may be chronic. This is an important distinction when choosing how to record the sway of the patient. In a case of acute imbalance, the patient may not be able to stand effectively and requires support. Although support can alter the amount that this person sways, studying the way in which the patient relies on different sensory cues, or how their balance changes over days and weeks, can lend insight into the central processes of recovery and contribute to the rehabilitation plan. Chronic imbalance might be a feature of postural phobic vertigo which is reflected by a better response to perturbations than when standing quietly, or when standing with their eyes closed compared to standing with eyes open (34, 35).

## Methodological considerations of posturography

Posturography is dependent on the availability of technical equipment to objectively capture postural instability. A force platform capable of measuring forces in different planes (preferably in anterior–posterior and mediolateral) at an appropriate sampling frequency and a package to analyse the forces produced by a person standing upon it is required. Although pre-built systems are available that sample force data, another approach is to use a customised platform where the sampling frequency can be adjusted depending on the suspected diagnosis or when different perturbations are used.

Although posturography is not diagnostic per se, customising the analysis could enhance its utility. An effective example is in patients with postural orthostatic tremor who generate more sway around 16 Hz (36). To capture this sway and be confident that it is not an artifact requires a longer recording. Another example is in Parkinson’s disease, in which patients sway can capture a Parkinsonian tremor between 4 and 7 Hz (37, 38). Current studies suggest that a Parkinsonian tremor can be reliably detected when the sampling frequency is set to 100 Hz (37, 38), a recording frequency that pre-built systems do not tend to record at. The advantage of adjusting the sampling frequency and the duration of recording can be seen when quantifying the proportion of postural sway that is at lower frequency and higher frequency (30). This is important because healthy participants have been shown to depend more highly on visual feedback when they express greater higher-frequency torque variance (a measure of the force exerted from the ankles and feet onto a surface) whilst standing on a foam surface (13).

The notion is that when an individual stands on a compliant foam pad, the mechanical properties of the foam - being its density, thickness and compliance - reduce the reliability of plantar cutaneous and proprioceptive input by absorbing and dispersing pressure signals (16, 39, 40). This reduction leads to decreased reliability of somatosensory cues used for fine-tuning postural adjustments. The CNS, facing a reduced quality and reliability of plantar somatosensory information, must consequently upweight vestibular and visual inputs to maintain postural control within appropriate limits (41). Critically, when foam posturography is performed with EC, the visual input is eliminated, forcing increased dependence on the vestibular system (12, 42). This paradigm thereby serves as an indirect probe of vestibular integrity by amplifying the postural instability arising from vestibular deficits.

Previous research from Patel and colleagues (32) and Gosselin and Fagan (43) have advanced the understanding of foam posturography by demonstrating that standing on foam does not only affect the quality and reliability of plantar somatosensory information. These investigations have revealed that the compliant nature of foam alters the mechanics of ankle and hip joint control, leading to modified motor strategies. Specifically, the foam surface impedes the ability to generate stable torque about the ankle. Instead, there is an increased reliance on proximal hip and knee musculature to maintain stability (13, 33). This biomechanical adaptation constitutes an essential element of the test’s sensitivity, as it taxes the sensorimotor integration pathways involved in postural control. Rather than serving solely as a sensory manipulation tool, the foam pad introduces a dynamic perturbation to the postural control system, challenging the CNS’s capacity to rapidly recalibrate motor outputs. Furthermore, cooling of the plantar sole has been shown to reduce somatosensory inputs from sway-sensitive mechanoreceptors and yields a different perturbation to standing on foam, further demonstrating that the perturbation caused by standing on foam extends beyond sensory dampening (32).

A compliant surface introduces both a temporal lag and a spatial distortion in the coupling between corrective ankle torque and the resultant restorative moment acting on the body’s centre of mass (13, 33). Paradoxically, a firmer foam, because it reacts more rapidly, may create a more mechanically unpredictable surface than a highly compliant foam that primarily absorbs energy and narrows the distance between the feet and solid surface beneath the foam pad (13, 33). This mechanical inefficiency precipitates a well-documented shift in postural strategy: from the simple ankle-driven inverted pendulum model to a multi-segment, hip-dominated control mode (33, 40). Thus, performance on foam does not merely reflect sensory processing capacity; it directly reflects the CNS’s ability to implement a state-dependent adaptation in its motor control algorithm. Maurer and colleagues (44) provide an elegant demonstration of this interaction, showing that foam surfaces degrade the spatial encoding of plantar pressure, forcing the postural control system to operate with an impaired afferent signal while simultaneously demanding a more complex efferent response.

Multiple studies have validated the theoretical framework of foam posturography. For instance, Patel and colleagues quantitatively demonstrated that anterior–posterior and mediolateral torque variance significantly increases when participants stand on foam compared to firm surfaces, especially with eyes closed (13). These findings confirm that foam surfaces effectively challenge both sensory feedback and motor control mechanisms involved in maintaining posture. Thus, foam posturography represents a multi-dimensional challenge to postural control, with a reduced quality and reliability of somatosensory input from the plantar surface of the feet and a mechanical obstruction of forces. The combination of these perturbing factors increases reliance on the vestibular system to control posture particularly when standing with eyes closed. Foam posturography could thus provide a sensitive and ecologically valid test of vestibular function.

A useful way to contextualise and summarise this is to contrast customised posturography with established clinical balance assessments, most notably CDP and its SOT, which remains the standard equipment in many balance clinics. CDP systems typically rely on fixed sampling frequencies (commonly 50–100 Hz), predefined trial durations and fixed outcome measures such as equilibrium scores and sensory ratios. While these metrics offer standardisation and are clinically interpretable, they constrain analytical processing. In contrast, customised posturography platforms allow sampling frequency, recording duration and signal processing to be tailored to the physiological functions of interest. This is particularly important for detecting pathological sway that occurs at higher frequencies. CDP systems do not typically include frequency-domain analysis of CoP or torque signals, limiting sensitivity to disease-specific oscillatory features. Although CDP provides a valuable, standardised framework for assessing balance impairment, its fixed protocols could limit sensitivity to condition-specific mechanisms of instability, particularly in early, stable or subtle vestibular disorders. Customised posturography, by contrast, offers a more nuanced interrogation of postural control mechanisms.

## Historical evolution and methodological variability in foam-based posturography

Foam posturography has evolved over several decades from a simple investigation into a quantitative and increasingly sophisticated tool for assessing postural control in vestibular disorders. The development has paralleled interest in sensory reweighting mechanisms and the need for accessible, low-cost alternatives to CDP. This section outlines the milestones and methodological differences in foam-based assessments, from bedside testing to laboratory-grade quantitative analyses.

### Bedside clinical tests: origins of the foam Romberg procedure

The clinical basis of foam posturography traces back to adaptations of the Romberg test, a classical neurological exam for detecting proprioceptive deficits. In its standard form, the Romberg test evaluates postural stability with eyes open (EO) and eyes closed (EC) on a firm surface, allowing clinicians to infer reliance on visual inputs when proprioceptive cues are compromised. However, the traditional Romberg test lacks sensitivity to explore vestibular dysfunction, as the somatosensory system delivers accurate information on firm surfaces, which largely compensates for vestibular loss (45). To address this limitation, clinicians began incorporating a foam pad under the feet to selectively affect the quality and reliability of somatosensory information from the plantar surface and ankle joints (39). When combined with eye closure, this “foam Romberg” test effectively changes two of the three primary balance inputs (visual and somatosensory), and thereby better display vestibular contributions to postural control. Patients with vestibular impairments often exhibit marked instability or frank falls under these conditions, whereas individuals with intact vestibular function are generally able to maintain stance for brief durations (12). These qualitative bedside applications, though widely used, are inherently limited by observer subjectivity, binary outcomes (fall vs. no fall) and inability to quantify subtle postural sway patterns. Nevertheless, the test remains a rapid, low-resource screening tool, particularly in acute care or rural settings where advanced instrumentation is unavailable. Importantly, foam Romberg testing may also aid in differentiating vestibular pathology from sensory neuropathy. In sensory neuropathy, instability may be more prominent on a firm surface with EC than on a foam pad with EO, due to profound proprioceptive loss (46).

### Quantitative static foam posturography: advancing objectivity and sensory profiling

Quantitative foam posturography paradigms employ laboratory-grade force plates or portable stabilometric systems to objectively measure balance control across multiple conditions. These systems track CoP trajectories over time, capturing kinematic data that reflects the integrated outputs from the vestibular, visual and somatosensory systems.

A standard protocol involves assessing postural sway under four sensory conditions:

1. Firm Surface, Eyes Open (Condition 1) – All sensory modalities are available; serves as the baseline.
2. Firm Surface, Eyes Closed (Condition 2) – Visual input is removed; reliance shifts to vestibular and somatosensory inputs.
3. Foam Surface, Eyes Open (Condition 3) – Somatosensory input is distorted; visual input remains available.
4. Foam Surface, Eyes Closed (Condition 4) – Both visual and somatosensory inputs are degraded; relies more on vestibular input.

Each condition is typically administered in 30–60 s trials, during which CoP metrics such as sway velocity (cm/s), sway path length, 95% confidence area of sway, sway frequency spectra, and anterior–posterior vs. medial-lateral displacement are recorded and analysed.

These parameters are further interpreted using sensory ratio indices, notably:

- Romberg Ratio (Condition 2/Condition 1) – Reflects reliance on visual input.
- Foam Ratio (Condition 4/Condition 3) – Reflects capacity to compensate for somatosensory degradation via vestibular input.
- Visual Ratio (Condition 4/Condition 2) – Reflects ability to substitute vestibular for visual input.
- Somatosensory Ratio (Condition 3/Condition 1) – Indicates effectiveness of vestibular/visual substitution for somatosensory inputs.

Such ratio analyses enhance the diagnostic specificity of foam posturography, distinguishing between isolated or combined sensory deficits and enabling subclinical detection of vestibular dysfunction, even when other vestibular tests (e.g., caloric testing or video head impulse test) yield equivocal (borderline) results (47).

## Review

### Key methodological studies and their contributions

Work by Fujimoto et al. (46), Lin et al. (48) and Liu and Kong (49) provided early validation of quantitative foam posturography. In a sample of 68 unilateral and 16 bilateral vestibulopathy patients, Fujimoto et al. observed significant elevations in CoP sway area, sway velocity and both Romberg and foam ratios compared to age-matched healthy controls (46). Most notably, the velocity-based Romberg ratio under foam EC yielded the highest area under the receiver-operating characteristic curve (ROC-AUC), highlighting its strong discriminative power for vestibular dysfunction.

Liu & Kong further contributed to methodological robustness by demonstrating excellent test–retest reliability of sway velocity across all four posturographic conditions in both patient and control groups (49). Their intraclass correlation coefficients (ICC range: 0.887–0.973) confirmed that when fall-inducing trials were excluded, foam posturography demonstrated high temporal stability and measurement precision supporting its utility in longitudinal rehabilitation tracking.

The addition of spectral sway analysis, which deconstructs sway signals into frequency bands, has further enhanced the interpretability of posturography data. Higher-frequency sway (typically >0.5 Hz) may reflect increased neuromuscular corrections and sensory conflict, while lower-frequency sway is associated with passive drift and may indicate delayed or ineffective compensatory strategies (30).

A summary of other key papers (78–81) is provided in the Appendix.

### Methodological heterogeneity and challenges

Interpretation of foam posturography data requires careful consideration of multiple methodological and physiological variables that can significantly impact test sensitivity, specificity and reproducibility. The importance of these factors was recognised in early posturography studies. For example, Kapteyn and colleagues (50) demonstrated that postural responses were highly dependent on test surface properties, stance configuration and trial duration in healthy individuals. More recently, Scoppa et al. (51) reinforced these observations, highlighting that methodological variability remains one of the primary barriers to cross-study comparisons. Variations to foam characteristics, test protocol parameters, instrumentation and subject population can introduce substantial heterogeneity in results across studies and clinical contexts. This section outlines the most critical factors affecting performance metrics in foam-based postural assessments.

Despite its promise, foam posturography remains methodologically heterogeneous, with substantial variation in protocols across studies. Key differences include:

1. Foam specifications: Foam thickness, density, and compliance vary widely, leading to differences to the degree of perturbation. Lack of standardisation severely limits data comparison.
2. Stance configuration: Protocols may vary between feet-apart, feet-together, semi-tandem, or tandem stance, each introducing differing degrees of postural challenge. These differences directly affect sway metrics and limit the establishment of universal thresholds.
3. Trial duration: Durations range from 10 to 120 s, with longer durations increasing sensitivity but also fatigue and fall risk.
4. Instrumentation sensitivity: Use of high-fidelity force plates versus low-resolution accelerometers leads to variability in metric sensitivity, especially for low-amplitude sway.
5. Outcome measures: Some studies report only binary outcomes (fall/no fall), while others provide continuous metrics (CoP velocity, area), impeding meta-analysis and clinical integration.
6. Population characteristics: Variation to participant age, diagnosis (acute vs. chronic), and comorbidities (e.g., Persistent Postural-Perceptual Dizziness (PPPD), diabetic neuropathy) influences sway behaviour and response to sensory perturbation.

These inconsistencies underscore the urgent need for protocol standardisation, normative dataset development and cross-centre calibration if foam posturography is to gain broader clinical acceptance.

#### Foam specifications: density, thickness, and material composition

The physical properties of the foam pad are central to the destabilising effect in foam posturography. Key parameters include foam density, thickness, compliance and material composition, all of which influence the degree of somatosensory perturbation during stance. Kapteyn and colleagues (50) originally showed that compliant surfaces disproportionately affect proprioceptive reliability thereby increasing reliance on vestibular inputs. Furthermore, studies by Patel and colleagues (13, 33) and subsequent layer variation experiments (52) have demonstrated that a foam with a higher elasticity modulus or foam thickness in the range of 4–5 cm (typically equivalent to 3–4 standard layers) optimally perturbs postural control while still permitting meaningful comparisons across individuals (13). Thinner pads (<2 cm) may be insufficient to induce measurable instability, while excessively thick foams (>6 cm) can result in intolerable imbalance, particularly in elderly or vestibular-impaired patients, increasing fall risk and confounding results due to premature trial termination.

**Figure 1:**
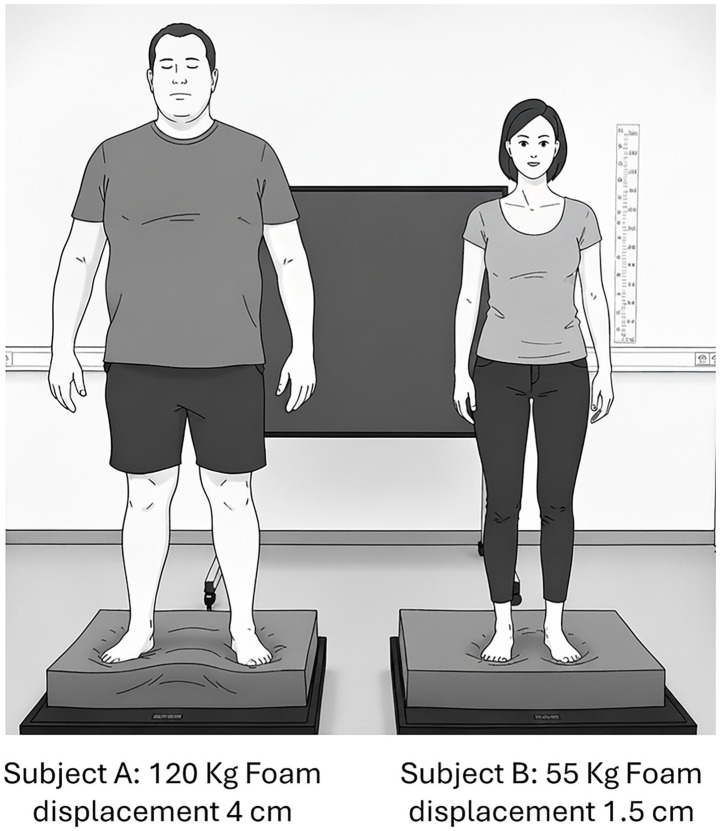
Illustration of how the same kind of foam may offer a different stability challenge depending on the test subject’s weight. Figure generated with AI: Nano Banana, Google AI studio.

Additionally, the foam density must balance rigidity and compliance. High-density foams may be so rigid as to effectively mimic firm ground, whereas ultra-low-density foams can behave unpredictably, producing non-linear CoP responses. Gosselin and Fagan emphasise that the foam–person interface constitutes a dynamic system subject to non-linear deformation (43), a concept aligned with the mass-dependent deformation effects observed by Kapteyn and colleagues (50). A high-mass individual may compress a foam pad beyond its linear elastic range, effectively “bottoming out” the material. This results in proprioceptive feedback from the rigid force plate beneath, fundamentally altering, and ultimately invalidating, the intended test condition, see Figure 1. These considerations point to the need for a load-matching paradigm in which the mechanical properties of the foam are appropriately scaled to the participant’s mass. Furthermore, Cohen and Sangi-Haghpeykar demonstrated that even minor variations in the density of foam can significantly affect performance. In their study, the denser foam was harder to stand on, underscoring the non-linear nature of postural challenge in foam-based assessments (53). Collectively, these findings suggest the existence of an optimal stiffness range that maximally taxes the postural control system. Despite these insights, no universal standard exists for foam material specifications, and variability between brands complicates reproducibility and inter-laboratory comparisons. This lack of standardisation remains a significant barrier to clinical adoption and normative data synthesis.

#### Stance configuration

**Figure 2:**
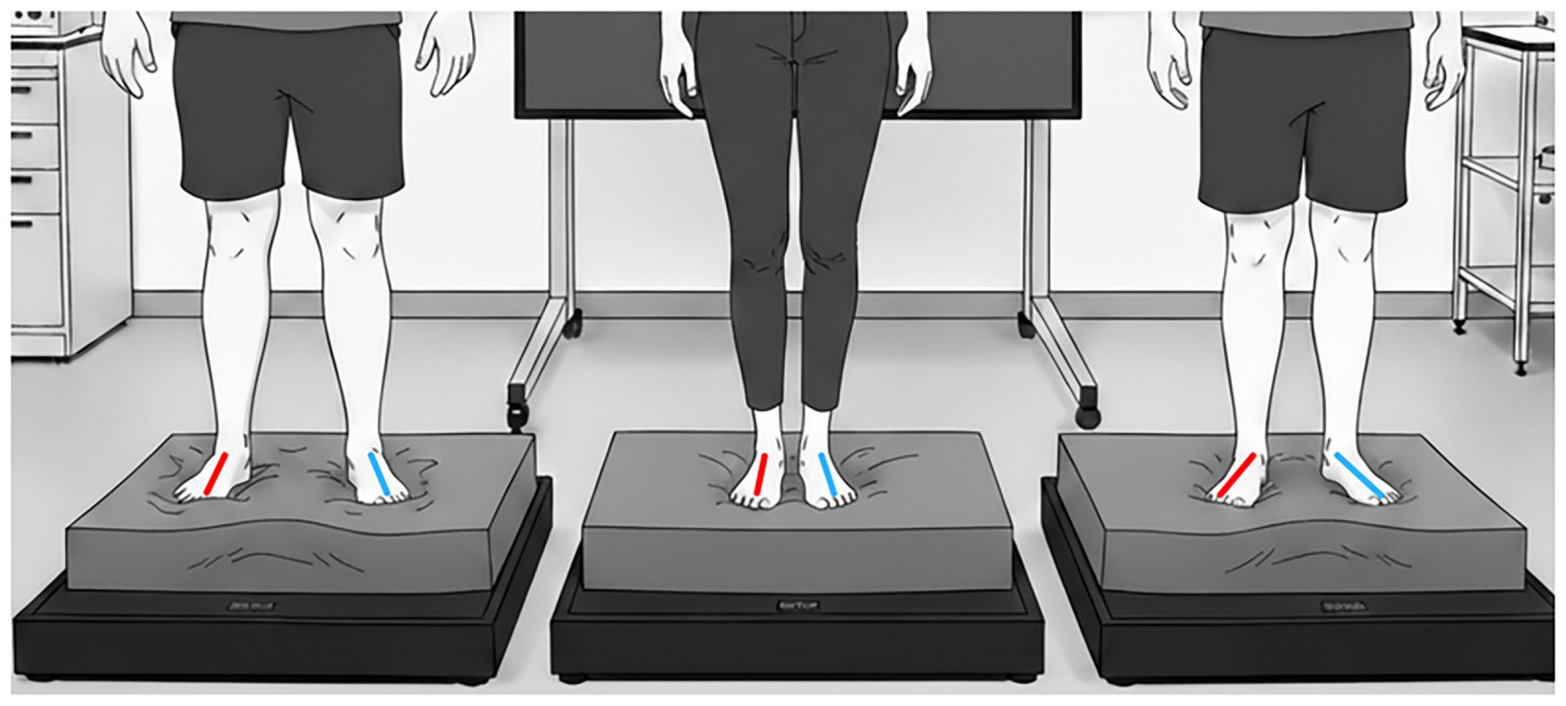
Illustration of different stance positions on the foam surface. The feet angles are marked with red and blue lines. From left to right: feet wide apart, feet close together, and heels together with toes apart. Figure generated with AI: Nano Banana, Google AI Studio.

Stance configuration, whether the feet are placed together, shoulder-width apart, or in a semi-tandem position affects the challenge imposed on postural control systems, see Figure 2. Narrower bases of support (e.g., feet together or tandem) reduce mechanical stability and thus amplifies the postural instability associated with standing with eyes closed or when the quality of somatosensory information is reduced (54). Consequently, the same foam surface may produce markedly different CoP profiles depending on stance width. Although feet-apart stance is generally better tolerated and safer in clinical populations, it may diminish the sensitivity to vestibular and somatosensory deficits, particularly in patients with chronic or compensated vestibular pathology. As a result, studies using differing stances cannot be directly compared without adjustment.

#### Trial duration

Trial duration and stance posture exert a profound influence on sway measurements and diagnostic sensitivity. The most frequently adopted durations in the literature range from 30 to 120 s per condition, with longer durations increasing sensitivity to subtle instability but also elevating the likelihood of participant fatigue or falls (12). Longer durations of test enhance the accuracy of sway frequency analysis and identifying subtle variations between control individuals and vestibular patients.

#### Instrumentation sensitivity: force plates vs portable devices

The choice of instrumentation significantly determines the clinical utility of posturography data. High-resolution force platforms such as those integrated into laboratory-grade posturographic systems offer detailed recordings of CoP trajectories, velocity, area, path length and spectral components of sway. These metrics provide quantitative data for assessing sensorimotor integration and disease-specific signatures (55). In contrast, portable stabilometry systems, including smartphone-based or inertial sensor-based devices, often rely on more limited parameters, such as maximum sway distance, standing time or binary fall/no-fall outcomes (56). While these tools are more accessible and suitable for fieldwork or low-resource settings, their sensitivity and specificity may be reduced, especially in detecting subclinical deficits or distinguishing between vestibular and non-vestibular sources of instability (57).

#### Outcome measures

Some clinicians may rely on qualitative observations or stopwatch-timed balance tests, whereas others use software-driven frequency domain analyses or stabilogram diffusion functions, providing greater diagnostic nuance. Thus, instrumentation and analysis heterogeneity are major limitations in standardising foam posturography across centres and studies, a challenge also pointed out by Duarte and Freitas (58).

#### Population characteristics: age, disease phase, and comorbidities

Physiological and clinical characteristics of the tested population have a substantial impact on posturography outcomes and their interpretation. For example, individuals with acute vestibulopathies (e.g., vestibular neuritis within days of onset) often display profound postural instability, particularly when standing on a foam pad with EC. However, some patients in the chronic phase of vestibular neuritis, especially those >3 months post-injury, may exhibit partial or near-complete compensation, with more nuanced techniques needed to detect underlying deficits.

Age is a particularly important modifier. Older adults exhibit naturally increased postural sway due to age-related declines in visual, somatosensory, and vestibular inputs, as well as reduced musculoskeletal responses (59). Normative data must therefore be stratified by age, with age-adjusted cutoffs used in clinical interpretation. However, such data remains limited, particularly for individuals over 80 years or children under 10 years, restricting widespread implementation of age-normed diagnostic thresholds (60).

Additionally, comorbidities and central neurological disorders can introduce confounding effects. For example, PPPD patients may show exaggerated sway due to maladaptive sensory weighting (e.g., visual dependence), while those with Ménière’s disease may demonstrate intermittent instability depending on disease phase or otolithic involvement (61).

Finally, studies have reported varying results in relation to sex, height, body mass index and foot size, although these are less frequently controlled for (43). Until comprehensive, population-specific normative datasets are developed, accounting for clinical diagnoses, disease chronicity and demographic factors, interpretation of foam posturography should be performed cautiously and contextually.

## Discussion

### Clinical utility of foam posturography in vestibular pathologies

Foam posturography has been increasingly utilised in clinical practice. Studies have examined its diagnostic performance, test–retest reliability, cost-effectiveness and pathophysiological relevance across a range of vestibular conditions. This section discusses the most compelling evidence on the clinical utility of foam-based postural testing, with emphasis on its diagnostic precision, reproducibility, affordability and sensitivity to specific vestibular subsystems.

#### Peripheral vestibulopathies: unilateral and bilateral

Fujimoto et al. (46) have investigated the clinical sensitivity of foam posturography in peripheral vestibular deficits (46). In their cohort study involving 68 patients with unilateral vestibulopathy (UVH) and 16 with bilateral vestibulopathy (BVH), postural sway metrics were evaluated against a control group of 66 healthy subjects under four standardised conditions: standing on firm and foam surfaces, with EO and EC. The study assessed eight CoP-based metrics, including mean sway velocity, sway area, and derived ratios such as the Romberg ratio (EC/EO on firm surface) and the foam ratio (foam/firm EO). Six metrics including CoP velocity and sway area were significantly elevated (*p* < 0.001) in both UVH and BVH patients relative to controls. The Romberg velocity ratio during the foam EC condition demonstrated the highest receiver operating characteristic (ROC) area under the curve (AUC), indicating excellent discriminative ability between affected and healthy individuals. In a subsequent analysis, Fujimoto’s group extended their findings to patients with chronic-stage UVH (>3 months post-onset). Even in the compensated phase, most sway parameters remained significantly elevated (greater postural instability) compared to controls (*p* < 0.01), providing strong evidence that impaired vestibulospinal postural responses may persist long after resolution of acute symptoms. This challenges the assumption that vestibular compensation fully restores balance and emphasises the role of continued rehabilitation for postural control.

#### Foam posturography in Ménière’s disease: utricular and otolithic insights

The role of the otolithic organs, particularly the utricle, in Ménière’s disease has garnered increasing attention, particularly due to the limitations of traditional caloric testing, which fails to adequately assess otolithic function. In this context, Lin et al. (62) conducted a novel study examining the relationship between foam posturography metrics and ocular vestibular-evoked myogenic potentials (oVEMP), a validated measure of utricular function (62). Results demonstrated that patients with abnormal oVEMPs had significantly higher Romberg area quotients compared to those with normal oVEMPs. This finding suggests that patients with utricular deficits are more reliant on vision to maintain balance, a compensatory mechanism consistent with central sensory reweighting. Although the AUC was not as high as in studies of UVH using sway velocity, the result is nonetheless clinically meaningful. The moderate correlation between oVEMP results and Romberg quotient implies that foam posturography is sensitive to subtle deficits in otolithic input, even in the absence of overt canal dysfunction. Importantly, sway area is influenced not only by dynamic instability but also by the magnitude of corrective strategies employed by patients, which may be greater in patients with reduced otolithic function. This study therefore supports the inclusion of foam posturography in the assessment of Ménière’s disease, particularly when oVEMP testing is unavailable or inconclusive. Furthermore, the elevated Romberg quotients suggest that Ménière’s disease patients with utricular involvement may benefit from vestibular rehabilitation that targets visual dependence and utricular adaptation.

#### Persistent postural-perceptual dizziness (PPPD)

Foam posturography has also been explored in the context of functional vestibular disorders, most notably Persistent Postural-Perceptual Dizziness (PPPD). This chronic condition, characterised by non-spinning dizziness, unsteadiness, and hypersensitivity to motion or visual complexity, is hypothesised to involve maladaptive central sensory processing rather than peripheral vestibular loss. In a recent study, Ichijo et al. (63) assessed 53 PPPD patients and age- and sex-matched healthy controls using foam posturography (63). They observed a consistent elevation in Romberg ratios, indicating an overreliance on visual input for balance. Simultaneously, foam ratios were decreased, suggesting underutilisation of somatosensory inputs even when available. Strikingly, these sensory imbalance patterns were evident even among PPPD patients with normal caloric tests, VEMPs, and vHIT, reinforcing the notion that central maladaptation, rather than peripheral dysfunction, is the principal driver of postural control abnormalities in PPPD. These findings underscore the value of foam posturography in capturing functional postural strategies and sensory weighting anomalies that elude traditional vestibular tests. Such insight is critical for designing personalised rehabilitation programs for PPPD, such as graded visual motion habituation, and retraining exercises that recalibrate sensory over-reliance.

### Interpretations and clinical implications

Foam posturography is increasingly recognised not only as a tool for assessing fall risk but also as a valuable instrument for understanding sensory integration, monitoring the response to therapy (e.g., gentamicin in Meniere’s disease) (64), and establishing differential diagnosis in patients with balance disorders. Its simplicity and accessibility contrast with more technologically demanding modalities, yet it offers unique insight into the interplay between visual, somatosensory, and vestibular contributions to postural control. Foam posturography has demonstrated high sensitivity in identifying postural control deficits associated with vestibular dysfunction, especially under conditions that challenge sensory reweighting such as standing on foam with EC. Notably, patients with subtle or compensated unilateral vestibular deficits may not have a pathological result on traditional vestibulo-ocular reflex (VOR) assessments, such as video Head Impulse Testing or sometimes in caloric irrigation (65). In contrast, foam posturography may reveal abnormal postural responses after recovery from the vestibular disorder (66, 67). This positions foam posturography as a valuable adjunct to standard vestibular diagnostics. When used alongside oculomotor tests, vestibular evoked myogenic potentials (VEMPs), and imaging, it provides a broader assessment of functional balance capacity, especially in conditions where subjective dizziness is present, but objective VOR measures are inconclusive. For example, foam-based sway metrics may reveal ongoing postural instability despite normal oculomotor findings.

#### Quantitative sensory weighting and clinical ratios

The utility of foam posturography is perhaps most effective when the results quantify sensory weighting strategies through derived ratios. For example, patients with PPPD often display an elevated Romberg ratio with minimal change in foam ratio, reflecting an overdependence on vision and underutilisation of somatosensory feedback (63). In contrast, patients with peripheral neuropathy may exhibit heightened sway across all foam conditions due to impaired proprioceptive input from the lower limbs with EO and EC (68–70), but standing with EC increases the contribution from the vestibular system (71).

The differential pattern of these ratios enables clinicians to distinguish between visual dependence, somatosensory loss and vestibular hypofunction, thereby tailoring rehabilitation approaches. Patients with high visual dependency may benefit from exercises reducing reliance on vision (e.g., EC balance training), whereas somatosensory impairment may require tactile enhancement or surface-specific retraining. Quantitative sensory weighting analysis thus offers a framework for rehabilitation planning and represents a significant advantage over qualitative balance assessments.

#### Monitoring rehabilitation and recovery

Foam posturography also serves as a longitudinal monitoring tool, enabling clinicians to track recovery and assess the effectiveness of vestibular rehabilitation interventions. Several studies have reported high test–retest reliability in sway parameters particularly in CoP velocity and sway area, across time points, making foam posturography suitable for serial assessments (48, 49). Moreover, tracking improvements in postural sway over multiple sessions allows clinicians to quantify patient progress, identify plateaus and adjust therapeutic intensity or modality accordingly. The ability to objectively document functional gains is particularly valuable in multidisciplinary care models. The low cost of foam posturography, portability and minimal setup requirements make it well-suited for use in outpatient vestibular clinics, tele-rehabilitation and even home-based rehabilitation programs.

#### Differential diagnosis across balance disorders

While many balance disorders share overlapping symptoms such as unsteadiness and fall risk, the underlying mechanisms differ substantially. Foam posturography provides a means to dissect these mechanisms based on performance under controlled sensory conditions. Patients with vestibular dysfunction typically perform well on a firm surface with EO and EC but experience marked instability on foam with EC (63, 72). In contrast, individuals with peripheral neuropathy, characterised by impaired proprioceptive feedback, exhibit poor balance on foam regardless of visual input (68–70). Similarly, patients with cerebellar ataxia often demonstrate increased sway even on firm surfaces, reflecting a central integrative deficit independent to sensory challenges (73, 74). Such patterns help clinicians determine which primary sensory deficit is causing the imbalance. Foam posturography provides clinically relevant information that is not given by conventional Romberg or tandem stance testing. It can also guide additional examinations (such as nerve conduction studies or neuroimaging) when results point to non-vestibular causes.

#### Utilisation of foam surfaces

Foam posturography fills a clinical need as it addresses limitations inherent in standard balance assessments. Bedside “push-pull” tests and the SOT primarily determine whether balance is maintained under altered sensory conditions but does not provide insight into how postural stability is achieved or which postural control strategies are used. As such, early or subtle deficits, such as maladaptive shifts from ankle-, knee- to hip- control, excessive high-frequency corrective activity or impaired adaptability may remain undetected despite preserved stance. Foam posturography enables quantification of frequency-specific sway and torque dynamics, allowing identification of pathological direction-specific behaviours and compensatory strategies that are analytically missed in commercial postural control analytics. Furthermore, whereas reflex-based vestibular tests assess peripheral pathway integrity in isolation, foam posturography with EC probes the vestibular contribution within a functional, whole-body task that simultaneously alters postural mechanics. By explicitly treating foam as a sensory manipulation, and also as a mechanical perturbation, foam posturography captures the CNS’s capacity for postural adaptation. When combined with appropriate sampling frequencies and analysis methods, foam posturography offers a pragmatic compromise between validity and quantitative rigour, enabling objective assessment of postural control when high-cost instrumentation is unavailable.

### Methodological evaluation - limitations and gaps in the literature

Although interest in foam posturography as a low-cost tool is growing, there are still significant methodological and practical limitations. Addressing these gaps through systematic research and consensus-based standardisation is essential to fully realise the clinical value of foam-based assessments.

#### Lack of standardisation in foam properties

A significant limitation across studies is the absence of standardised specifications for the foam surfaces employed. The destabilising effect of foam in posturography is governed by several characteristics, each of which influences postural responses through distinct mechanisms. The most relevant foam properties include thickness, density, compliance and material composition. Investigations have utilised foam pads of varying thicknesses (ranging from 2 cm to 10 cm), densities, and material compositions, ranging from open-cell polyurethane to viscoelastic memory foams, without reporting mechanical properties such as compliance, damping coefficients or the elastic modulus. The thickness of foam is a crucial property that determines the extent of somatosensory distortion and mechanical impedance at the foot–surface interface. Increasing foam thickness generally increases instability. Density and compliance (often measured as the elastic modulus) jointly determine how the foam deforms under body weight. Importantly, foam deformation is non-linear and load-dependent where heavier individuals may compress compliant foam beyond its linear elastic range. Material composition (e.g., polyurethane vs. viscoelastic memory foam) further modulates these effects. Viscoelastic materials exhibit time-dependent deformation and recovery, meaning that postural responses may change over the course of a trial as the foam progressively deforms and adapts to the load. This introduces additional variability and may obscure deficits in postural control, particularly in longer trials. Collectively, these characteristics directly influence the extent to which vestibular contributions to balance are evidenced and how impairment is presented. Establishing consensus recommendations for foam mechanical properties, linked to participant mass or load-response characteristics, would represent a critical step toward standardising foam-based posturography protocols.

Given that the biomechanical challenge posed by the foam is the central variable in these assessments, such heterogeneity prevents the replication of studies and precludes meta-analysis (53). Without a standardised foam type and configuration, it is not possible to develop consistent diagnostic thresholds or normative benchmarks for sway metrics. As a result, clinicians and researchers lack a firm basis upon which to interpret patient results or compare findings across populations. This problem is exacerbated by the absence of regulatory guidance or consensus protocols from professional bodies.

#### Fall censoring bias and ecological validity

A methodological challenge of many foam posturography studies is the issue of fall censoring. To ensure test safety and protect participants from injury, most studies either terminate trials once instability leads to stepping or falling, or they exclude patients deemed too unstable to perform foam-based trials safely. While this is ethically necessary, such exclusion introduces a censoring bias that potentially underestimates the true extent of postural instability in vestibular populations, especially those at highest risk for real-world falls. By systematically removing data from fall-prone individuals, researchers risk mischaracterising the distribution of sway metrics and overestimating average postural performance. This has important implications for both the sensitivity and ecological validity of the test. Future research should aim to develop adapted protocols that can capture instability without excluding fall-prone patients, such as using harnessed balance platforms or data extractions, to mitigate this bias and improve the generalisability of findings to clinical assessment of fall risk.

#### Sample sizes and heterogeneous study populations

Many studies with foam posturography rely on relatively small sample sizes, often with fewer than 30 patients per diagnostic group, and utilise heterogeneous inclusion criteria that encompass a broad range of vestibular disorders, disease durations and comorbidities. This methodological heterogeneity impairs the statistical power to detect subtle differences in sway parameters, particularly in subgroups such as early-stage unilateral vestibulopathy. Moreover, pooling data across diagnostically diverse patient populations complicates the interpretation of findings, as distinct vestibular syndromes may exhibit unique patterns of sensory compensation and postural adaptation. Without adequately powered and stratified analyses, foam posturography may not be fully utilised to inform differential diagnosis or individualised rehabilitation.

#### Limited normative data across age, stance, and diagnosis

The interpretability of foam posturography remains severely limited by the absence of large-scale normative data across key demographic and clinical populations. Current reference values are derived from small, age-restricted samples, typically composed of healthy young adults. There is a critical need for normative data that encompass a wider age range including paediatric, middle-aged, and older adult populations and account for age-related changes in proprioception, vestibular function and postural control strategies. Moreover, normative values should be stratified by foot stance (e.g., Romberg vs. tandem), as well as by test condition (e.g., EO vs. EC, firm vs. foam surface). This is necessary to derive age-appropriate cutoffs for abnormal sway, develop percentile-based reporting systems and calibrate risk thresholds for clinical populations. Importantly, the development of normative values must also account for different posturographic parameters. As discussed by Paillard and Noé (75), posturographic measures such as sway area, path length, and velocity exhibit variability and inconsistent responsiveness across populations when task demands and conditions vary. Without consensus on which postural parameters offer the greatest sensitivity to disease and robustness, normative thresholds risk being misleading. To summarise, disease-specific normative comparisons are needed to refine diagnostic sensitivity in conditions. Until such data are available, clinicians must interpret foam posturography results with caution, relying on relative comparisons or within-subject longitudinal trends rather than absolute thresholds.

#### Inconsistent testing protocols

A further obstacle to the clinical adoption of foam posturography is the lack of protocol standardisation across research studies. Variations in trial duration, foot stance, visual conditions and instrumentation (force plates vs. inertial measurement units) lead to substantial variability in measured outcomes. Even small changes in stance width or trial duration can significantly affect CoP velocity and sway area, making it difficult to interpret findings or apply them to clinical practice. Standardised testing protocols are urgently needed to facilitate cross-study comparisons, enable normative benchmarking and enhance reliability. Protocol elements such as foam type, foot positioning, trial length, instructions to participants (e.g., “stand naturally” vs. “stand as still as possible”), and exclusion criteria must be consistent if foam posturography is to transition from research to routine vestibular diagnostics.

#### Foam thickness optimisation in posturography

In a controlled investigation, Liu and colleagues explored how changing the thickness of the foam pad affected the sensitivity and specificity of posturography testing in UVH (52). The study was driven by the understanding that foam pads degrade somatosensory input by introducing compliance and instability to the base of support. However, there exists an important trade-off, while increased thickness may enhance the challenge to proprioception, it can also lead to an increased rate of falls or compensatory stepping, as distance between the feet and firm surface beneath the foam pad increases, thereby limiting clinical utility.

Participants stood on foam composed of 1 to 5 stacked layers, each approximately 1 cm thick. The test was conducted with both EO and EC for each foam thickness. CoP velocity and standing time were recorded as primary outcome measures. Healthy controls and UVH patients were included for comparative analysis. The results showed a linear relationship between foam thickness and postural instability, as sway velocity increased progressively with each added layer of foam, with both EO and EC. Critically, while both investigational groups exhibited increased sway with thicker foam, UVH patients showed a disproportionately greater deterioration in postural control with EC, particularly with 4- and 5-layers of foam. Additionally, the average standing time prior to loss of balance (defined as stepping, stumbling or falling) decreased markedly in UVH patients as foam thickness increased. These effects were most pronounced at the 4-layer level, where the difference between UVH patients and controls for sway velocity was highest.

To quantify the diagnostic efficacy of these conditions, ROC curve analysis was performed. The ROC for sway velocity in the 4-layer foam EC condition yielded an AUC of 0.90, demonstrating excellent diagnostic discrimination. Sensitivity and specificity at this thickness were approximately 85 and 87% respectively, suggesting that this configuration strikes the optimal balance between challenge and clinical feasibility. The study’s findings have direct implications for standardising foam posturography protocols. Specifically, the 4-layer foam pad with EC appears to be the most sensitive and specific configuration for distinguishing UVH from healthy balance. Using thinner foam may not adequately destabilise posture, while thicker configurations risk inducing early termination due to falls.

#### Longitudinal and multicentre outcome trials

Longitudinal cohort studies are required to evaluate how foam-based metrics change over time in patients undergoing vestibular rehabilitation or pharmacologic treatment. These studies should aim to establish

1. Minimal detectable change: the smallest change in sway velocity or area that exceeds test–retest variability.
2. Minimal clinically important difference: the smallest change associated with a perceived or functional improvement in balance.
3. Predictive validity: the extent to which baseline posturography metrics predict clinically relevant outcomes such as falls, return to work or functional independence.

Ideally, such investigations should be multicentre in nature to ensure sufficient sample sizes and external validity.

#### Reliability and reproducibility of posturographic measures

For a clinical balance assessment tool to be considered robust, high test–retest reliability is essential. To explore this, Liu and Kong (49) conducted important studies evaluating the reproducibility of foam posturography metrics in both patients with vertigo and healthy controls. Across repeated sessions under multiple test conditions, Liu and Kong reported intraclass correlation coefficients (ICCs) ranging from 0.887 to 0.973 for sway velocity. These values fall within the range considered “excellent reliability” by established psychometric standards, suggesting that foam posturography yields consistent and reproducible results when performed under standardised protocols. Importantly, trials in which participants lost balance or stepped were excluded to avoid statistical artifacts introduced by fall-related variance. This exclusion, while methodologically sound for evaluating reliability, also highlights the need for protocols that can safely include fall-prone individuals in future research to ensure broader applicability.

#### Comparative and cost-effectiveness analyses

Although CDP remains the gold standard for comprehensive balance testing, it is costly and often inaccessible in resource-limited settings. Several studies have evaluated foam posturography as a cost-effective alternative. Enache et al. (76) directly compared traditional foam-based posturography with the SOT component of CDP in a population of patients with diagnosed peripheral vestibular dysfunction. They found that patients exhibited significantly increased CoP sway in foam conditions (*p* < 0.05), particularly during the foam EC task. The results correlated strongly with SOT scores, supporting the view that foam posturography captures similar postural deficits while circumventing the need for expensive, mechanised platforms. Celebisoy and colleagues echoed these findings in a policy-oriented publication, advocating for the widespread use of foam posturography in countries where healthcare budgets and equipment access may limit routine CDP availability (77). Celebisoy and colleagues argued that foam-based testing offers a scalable, low-barrier tool for screening and rehabilitation, particularly in primary care or community-based vestibular clinics. Collectively, these studies position foam posturography as a feasible, economical alternative to CDP, enabling broader access to balance diagnostics across wide healthcare environments.

## Conclusion

Foam posturography is a robust, valid, reliable and cost-effective tool that specifically challenges somatosensory input to increase demands on the vestibular system. It can yield objective measures that assist in determining a vestibular impairment and monitoring the results of rehabilitation. While inconsistencies in methodology and normative benchmarks limit universal adoption, its low cost and portability make it a pragmatic adjunct in clinical vestibular assessment and research.
