# Supplementary material for: Critical evaluation of the benefits and limitations of foam posturography in vestibular disorders: a narrative review
Source: Front Neurol. 2026 Feb 25;17:1771719. doi: 10.3389/fneur.2026.1771719 (PMC12975558; doi:10.3389/fneur.2026.1771719)
Supplement: Supplementary file 1 [file Data_Sheet_1.pdf]

Appendix

Summary of key papers

Fujimoto et al 2013 Factors affecting postural instability in Meniere's disease (78). Chisato Fujimoto, Naoya Egami, Makoto Kinoshita, Keiko Sugasawa, Tatsuya Yamasoba, Shinichi Iwasaki

Fujimoto and colleagues investigated the effectiveness of foam posturography in patients with definite unilateral or bilateral Meniere's disease and divided patients into three groups based on disease duration. Bipedal stance was assessed with eyes open and eyes closed in 54 patients with definite Meniere's disease and a healthy control group on a 5cm thick foam block with a density of 0.162 g/cm^2. An increased Romberg's ratio (Eyes closed/Eyes open) of CoP velocity on a foam surface significantly correlated to a weaker response on caloric testing and/or cVEMP testing, but not on a firm surface. There was no effect of disease duration. Patients with a pathological caloric and/or a pathological cVEMP had an increased postural sway velocity when standing on foam for the Romberg's ratio of CoP velocity, Romberg's ratio of CoP area and CoP area on foam with eyes closed compared to a firm surface. CoP area on foam was more sensitive to Meniere's disease vs. the firm surface, shown using a firm surface/foam surface quotient.

Fujimoto et al 2014. Postural stability in vestibular neuritis: age, disease duration, and residual vestibular function (79). Chisato Fujimoto, Naoya Egami, Makoto Kinoshita, Keiko Sugasawa, Tatsuya Yamasoba, Shinichi Iwasaki

Fujimoto and colleagues retrospectively investigated the factors that affect foam posturography in patients with vestibular neuritis in either the acute or chronic phase. Patients were divided into four groups based on the onset of symptoms: 1. 10 days or fewer, 2. 11-30 days, 3. 31-90 days, and 4. 90 days or longer. Bipedal stance was retrospectively assessed with eyes open and eyes closed in 58 patients, and a healthy control group on a 5cm thick foam block with a density of 0.162 g/cm^2. Postural instability was higher in patients with vestibular neuritis compared to controls. A disease duration of 10 days or fewer, the degree of caloric paresis and increased age all contributed to greater postural instability. Romberg's ratio for CoP velocity (Eyes closed/Eyes open) with foam was more sensitive to vestibular neuritis than other tests.

Fujimoto et al 2013. Effect of severity of vestibular dysfunction on postural instability in idiopathic bilateral vestibulopathy (80). Chisato Fujimoto, Toshihisa Murofushi, Yasuhiro Chihara, Munetaka Ushio, Mitsuya Suzuki, Takuhiro Yamaguchi, Tatsuya Yamasoba, Shinichi Iwasaki

Fujimoto and colleagues retrospectively investigated the effectiveness of foam posturography in patients with idiopathic bilateral vestibulopathy. Patients were divided into three groups: 1. Patients who showed abnormal caloric responses in the presence of normal cVEMPs bilaterally (superior type), 2. Patients who showed no cVEMP responses in the presence of normal caloric responses bilaterally (inferior type), and 3. Patients who were in neither of these categories had both abnormal caloric and cVEMP responses. Bipedal stance was retrospectively assessed with eyes open and eyes closed in 29 patients who fulfilled criteria, and a healthy control group on a 5cm thick foam block with a density of 0.162 g/cm^2. Postural instability was higher in the patients with bilateral vestibulopathy compared to controls, apart from patients with a pathological cVEMP bilaterally and normal caloric test bilaterally with eyes closed on a foam surface. The Romberg's ratio (Eyes closed/Eyes open) of CoP velocity with foam was more sensitive to bilateral vestibular loss than other tests.

Fujimoto et al 2010 Effects of unilateral dysfunction of the inferior vestibular nerve system on postural stability (81). Chisato Fujimoto, Toshihisa Murofushi, Yasuhiro Chihara, Munetaka Ushio, Takuhiro Yamaguchi, Tatsuya Yamasoba, Shinichi Iwasaki

Fujimoto and colleagues investigated the effectiveness of foam posturography in the chronic phase following unilateral peripheral vestibulopathy, as confirmed in clinical presentation and with vestibular hypofunction in the affected ear with cervical vestibular evoked myogenic potentials (cVEMP) and/or the bithermal caloric test. The loss of function was attributed to either unilateral vestibular neuritis, acoustic neuroma, Meniere's disease, sudden deafness with vestibular loss, endolymphatic hydrops, otosclerosis or of unknown pathologies. Bipedal stance was assessed with eyes open and eyes closed in 108 patients and a healthy control group on a 5cm thick foam block with a density of 0.162 g/cm^2. Results were that unilateral vestibular pathology increased postural instability using the Romberg's ratio (Eyes closed/Eyes open), CoP velocity and CoP area. A firm surface/foam surface quotient showed that the foam block was not required to distinguish patients from controls in the eyes closed test. The Romberg's ratio of CoP area significantly distinguished patients with a pathological cVEMP and caloric test from patients with a pathological caloric test only.

Fujimoto et al 2012 Assessment of postural stability using foam posturography at the chronic stage after acute unilateral peripheral vestibular dysfunction (72). Chisato Fujimoto, Toshihisa Murofushi, Keiko Sugasawa, Yasuhiro Chihara, Muentaka Ushio, Tatsuya Yamasoba, Shinichi Iwasaki

Fujimoto and colleagues investigated the effectiveness of foam posturography several months (after 3 months) after an acute unilateral peripheral vestibulopathy in the acute phase and the chronic phase, as confirmed in clinical presentation and with vestibular hypofunction in the affected ear in both the acute and chronic stages with the bithermal caloric test. The loss of function was attributed to either unilateral vestibular neuritis, sudden deafness with vestibular loss or surgical ablation of the vestibulocochlear nerve. Bipedal stance was assessed with eyes open and eyes closed in 34 patients and a healthy control group on a 5cm thick foam block with a density of 0.162 g/cm^2. Results were that unilateral vestibular pathology increased postural instability at both the acute and chronic stages using the Romberg's ratio (Eyes closed/Eyes open), CoP velocity and CoP area. A firm surface/foam surface quotient showed that the foam block was not required to distinguish patients from controls in the eyes closed test. The Romberg's ratio of CoP area significantly distinguished patients in the acute phase from the chronic phase.

Liu et al 2018. Foam pad of appropriate thickness can improve diagnostic value of foam posturography in detecting postural instability (52). Bo Liu, Yangming Leng,Renhong Zhou, Jingjing Liu, Dongdong Liu, Jia Liu, Su-Lin Zhang, Wei-Jia Kong

The study investigated how foam-pad thickness influences the diagnostic sensitivity of foam posturography in detecting postural instability in 33 patients with unilateral vestibular hypofunction, comprising 15 with definite Ménière's disease, 14 with idiopathic sudden deafness with vestibular impairment, and 4 with vestibular neuritis, compared with 30 healthy controls. Participants stood on firm ground and on 1–5 layers of foam, each layer 2.54 cm thick with density 44.8 g/cm^3 and compliance 0.086 kPa^{-1}, while sway velocity (SV), standing time before falling, and fall reactions were recorded. SV increased as foam thickness increased, but no further increase occurred between 4 and 5 layers, indicating a saturation plateau, consistent with prior findings that postural parameters level off as surface compliance rises. The authors highlight that foam properties such as density and elastic modulus, previously shown to influence torque variance and instability, also shape postural responses. In the patient group, standing time under eyes-closed conditions decreased progressively with increasing foam thickness, yet sway did not worsen from 4 to 5 layers. ROC analyses showed

that 4-layer foam with eyes closed provided the best discrimination between vestibular-impaired patients and controls. The authors concluded that foam of appropriate thickness and mechanical characteristics and recommend that foam-posturography protocols are adjusted according to the specific density, elasticity, and compliance of their foam pads.
